# Empirical versus preemptive antimycotic therapy in terms of outcome benefit

**DOI:** 10.1186/cc12022

**Published:** 2013-03-19

**Authors:** S Milanov, VT Todorova, G Georgiev, M Milanov

**Affiliations:** 1 Emergency Hospital 'Pirogov', Sofia, Bulgaria

## Introduction

Novel treatment strategies for invasive candidiasis (IC) are constantly emerging. Nevertheless, difficulties in diagnosis pose a challenge on their reliability, efficacy and safety. We have previously developed and approbated in our ICU an algorithm for empirical antimycotic therapy, combining the most significant risk factors for IC with three major clinical criteria for persistent nonbacterial sepsis [1]. On the other hand, preemptive therapy, based on identification of mycotic antigens and/or anti-mycotic antibodies in serum, is regarded as more reliable, even though it is known for its low sensitivity. The aim of the current study was to compare and evaluate the possible outcome benefit of our protocol implementation versus detection of galactomanan in patient's serum as a trigger for antimycotic treatment initiation.

## Methods

A randomized prospective controlled trial was carried out from September 2010 to September 2012. After the implication of the inclusion and exclusion criteria, patients were submitted to block randomization and stratified on the basis of their initial SAPS II exp score. Antimycotic therapy was started on the day of inclusion in the control group and only with positive galactomanan serum test in the preemptive therapy group. Initial data were gathered on demographics, proven risk factors for IC-related mortality, severity of inflammatory response and organ dysfunction. Dynamics of SIRS and SOFA values, Candida colonization index, ventilator-free days, length of ICU stay and outcome were followed for each patient.

## Results

A total of 106 patients were enrolled. No statistically significant differences in their basal characteristics were found. The subsequent SIRS and SOFA scores showed firm dynamics in the control group, although the new organ dysfunction severity was insignificantly lower. The length of ICU stay and the number of ventilator-free days were comparable. The in-hospital mortality was 47.1% in the preemptive therapy group versus 31.3% in the control group (*P *= 0.94). A total of seven adverse reactions were observed among treated patients, yet not associated with higher mortality risk.

## Conclusion

The choice of empirical versus preemptive therapy led to earlier and more stable reduction in the degree of organ dysfunction severity. It showed to be at least not inferior if not equal; in terms of survival benefit and expediency of treatment. Moreover, galactomanan detection fails to guide the choice of the individual antimycotic, based on the expected Candida spp.
